# Performance of a Yeast-mediated Biological Fuel Cell

**DOI:** 10.3390/ijms9101893

**Published:** 2008-10-08

**Authors:** Anuradh Gunawardena, Sandun Fernando, Filip To

**Affiliations:** 1 Department of Biological and Agricultural Engineering, Texas A&M University, College Station, TX 77843, USA; 2 Department of Agricultural and Biological Engineering, Mississippi State University, MS 39762, USA

**Keywords:** Fuel cell, yeast, mediators, bio-catalyst

## Abstract

*Saccharomyces cerevisiae* present in common Baker’s yeast was used in a microbial fuel cell in which glucose was the carbon source. Methylene blue was used as the electronophore in the anode compartment, while potassium ferricyanide and methylene blue were tested as electron acceptors in the cathode compartment. Microbes in a mediator-free environment were used as the control. The experiment was performed in both open and closed circuit configurations under different loads ranging from 100 kΩ to 400Ω. The eukaryotic *S. cerevisiae*-based fuel cell showed improved performance when methylene blue and ferricyanide were used as electron mediators, rendering a maximum power generation of 146.71±7.7 mW/m^3^. The fuel cell generated a maximum open circuit voltage of 383.6±1.5 mV and recorded a maximum efficiency of 28±1.8 % under 100 kΩ of external load.

## 1. Introduction

Due to increasing energy prices and concerns on long-term energy security, there is a renewed interest in bio-renewable energy generation technologies. Consequently, direct electricity generation from glucose, a bio-renewable feedstock, has regained momentum. This study is an in-depth look at the behavior of a fuel cell using Baker’s yeast (*Saccharomyces cerevisiae*) in a proton exchange membrane (PEM) microbial fuel cell (MFC). Yeast based fuel cells are of interest since these could be easily retrofitted into ethanol plants for in situ power generation.

**Figure 1.**
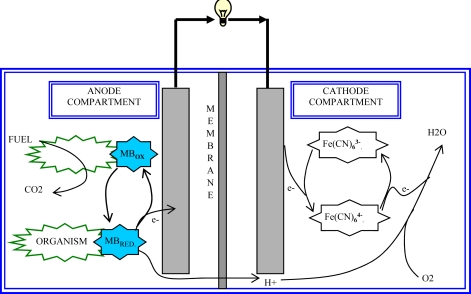
The yeast fuel cell with its constituent redox cycles in the anode and the cathode compartment. Note: MB_ox_ – Methylene Blue (oxidized); MB_RED_-Methylene Blue (reduced)

The function of microbes in a fuel cell is to catalyze the reaction that involves conversion of chemical energy into electrical energy [1–3]. The metabolic processes of these microorganisms produce electrons by oxidizing a carbon source, which in many applications, is a carbohydrate monomer. The electrons generated at the anode can then be passed through an external circuit to produce power. These electrons enter the cathode to combine with the protons (H^+^) that transfer through a PEM and bind with externally provided oxygen to form water (Figure 1).

Presently, MFC research is highly focused on wastewater treatment [4–6]. The organic sludge is used as the carbon source for organisms to oxidize. To date, different organisms have been experimented in MFCs and depending on the need for presence or absence of mediators to complete the redox reactions, two main types of fuel cells have been documented: (i) Fuel cells with mediated electron transfer and (ii) Fuel cells with direct electron transfer [7].

In fuel cells with mediated electron transfer, an intermediate molecule, preferably a dye, will shuttle electrons between the microbe and the electrode. The staining ability of a dye helps it to stick to the cellular membrane and helps transfer of electrons and protons. Direct electron transfer is now a research interest where the organisms posses metal complexes on its membrane making exogenous mediators unnecessary [8–11]. Indigenous mediators reduce the risk of poisoning the medium, which is considered a serious drawback of the exogenous mediators. Commonly used organisms in direct electron transfer fuel cells are *Shewanella putrefaciens, Geobacter sulfurreducens, Geobacter metallireducens* and *Rhodoferaxferrireducens* [12–14]. However, in Baker’s yeast-based fuel cells, exogenous mediators are a necessity since *S. cerevisiaes* is not known to produce such mediators indigenously. Tests with organisms capable of generating mediators have proved higher conversion efficiencies compared to other MFCs with mediators which transports electrons between the species and the electrode. However, the conversion efficiency itself is inadequate to determine the fuel cell behavior as it does not sufficiently explain the energy generation mechanisms in the cell. Therefore, additional parameters such as: (i) metabolism of the bacterial species, (ii) electron transfer rate of the microorganisms, (iii) the effectiveness of the proton exchange membrane, and (iv) internal resistance have to be considered for effective performance analysis of a fuel cell. This work, in part, attempts to fulfill this knowledge gap using *S. cerevisiae* as the model microorganism.

The presence of oxygen in the anode compartment is not favorable for the overall fuel cell function. Oxygen is the terminal electron acceptor in the cathode compartment and therefore, the presence of it in the anode compartment would disrupt the electron flow through the external circuit. This obliges an oxygen free environment in the anode compartment of the MFC. *S. cerevisiae* like many other microorganisms can function in anaerobic conditions. It is an easily accessible microorganism with a well understood metabolism. The growth of *S. cerevisiae* is optimum at the ambient temperature which is around 30 °C. These factors prompted us to choose *S. cerevisiae* for this study.

**Figure 2.**
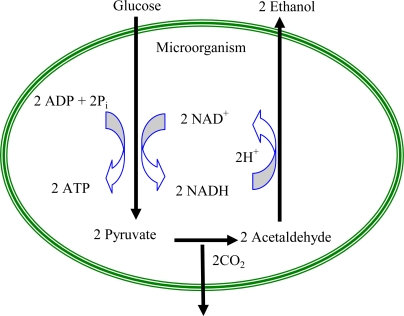
Anaerobic fermentation pathway of *Saccharomyces cerevisiae* - under anaerobic conditions, yeast will transform pyruvate to ethanol. The reduction of NAD+ to NADH will generate two ATP molecules, two H+ ions and two electrons [15].

Under anaerobic conditions, as shown in the Figure 2, this organism switches to a fermentation reaction where two molecules of pyruvate are produced from one glucose molecule. Pyruvate will be further transformed to two molecules of acetaldehyde by the enzyme pyruvate decarboxylase. Acetaldehyde thereafter will be transformed to alcohol by alcohol dehydrogenase, which is an NADH dependent enzyme. In anaerobic fermentation the recycling of NADH to NAD^+^ is important to keep the glycolysis process continuous [15, 16]. Since this glycolysis reaction takes place in the cytosol of the cell rather than in the mitochondria, NADH is easily accessible to a mediator molecule that is attached to the cell membrane. The MFC which operates using *S. cerevisiae* extracts the energy using NADH/NAD^+^ redox cycle. The organism can sustain life as long as the glycolysis pathway is not obstructed. The energy extraction process in the fuel cell does not disrupt the glycolysis as NADH is oxidized back to NAD^+^ when a mediator gets reduced.

The main objective of this study was to parameterize the performance of a *Saccharomyces cerevisiae* based fuel cell. The effect of extraneous mediators on open circuit voltage, current and power under different loads, the internal resistance and the efficiency were determined.

## 2. Materials and Method

### 2.1. Construction of the Fuel Cell

The fuel cell chamber was constructed from PVC and the internal diameter of the chambers was 5 cm in diameter and each compartment had a volume of 500 mL. The two chambers were separated by a Proton Exchange Membrane (PEM) where the membrane was held by a coupling between the chambers. A DuPont Nafion 117 PEM was purchased from the Ion Power Co. and each experiment was performed with a new membrane to avoid any interferences and/or contaminations from previous experiments. The electrodes were 45 PPI (Pores per Inch) Reticulated Vitreous (RV) carbon, with dimensions 1”× 1” × 6” purchased from ERG (Oakland, CA). Copper (Cu) wires were soldered to the electrodes by using a conductive epoxy. A digital pH transmitter (Sensorex, PHMA transmitter and pH probe) was used to measure pH variations during experimentation. The IoTech WaveBook-12 with a sampling rate of 0.1Hz was used as the automated data acquisition system.

### 2.2. Operation of the Fuel Cell

Cell growth measurements were not observed in order to minimize oxygen contamination to the fuel cell as well as to keep liquid volumes in the anode compartment static during experiments. On each operation, the fuel cell compartments were cleaned and the electrodes were autoclaved for 30 min. All the substrates, i.e., *Saccharomyces cerevisiae*, methylene blue (MB), potassium ferricyanide (PF) and D-glucose were purchased from Sigma Aldrich. The concentration of both MB and PF solutions were 50 mM. The anode solution was prepared with 2 g of yeast and 0.12M D-glucose which was prepared in either MB or de-ionized water — stirred well using a magnetic stirrer. In the experiment, the combinations of the solutions were selected according to Table 1. MB was tested as the anode mediator and on the cathode side both MB and PF were tested as both have the property of being reduced by oxygen. After a new batch was fed, both anode and cathode compartments were sparged with 300 mL/min CO_2_ and O_2_ respectively. The open circuit voltage (OCV) was measured continuously using the automated data acquisition system. A completely randomized design (CRD) was used in conducting the experiment and the data were analyzed using a statistical analytical software SAS. All the inferences were based at P = 0.05 significance level. The data points and the values are represented with ± standard error.

## 3. Results and Discussion

### 3.1. Variation of Open Circuit Voltage

**Figure 3.**
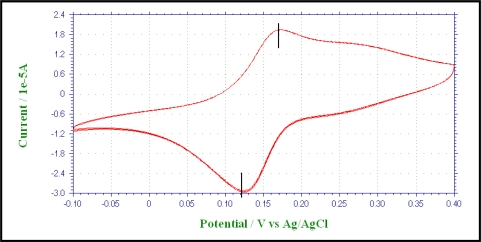
The cyclic voltammogram for 50 mM methylene blue solution with carbon electrode. Scan rate 0.05 V/s, potential range –0.1 V– 0.4 V. Reduction and oxidation peaks at 0.13 V and 0.18 V respectively.

**Figure 4.**
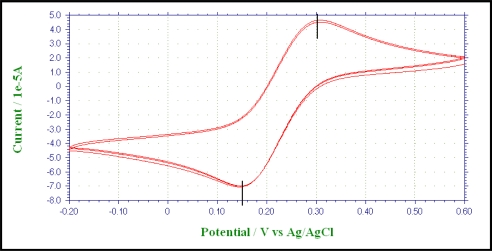
The cyclic voltammogram for 50 mM potassium ferricyanide solution with carbon electrode. Scan rate 0.05 V/s and the potential range –0.2 V to 0.6 V. Reduction and oxidation peaks at 0.13 V and 0.20 V respectively.

During the first phase of the experiment, a cyclic voltametry (CV) study was conducted to understand the reversible nature of the methylene blue (MB) and potassium ferricynanide (PF). Since MB is not toxic for the microorganism, it can be used as the mediator in the anode compartment - provided it is reversible between the reduced and the oxidized states. According to Figure 3, it can be explained that MB is reversible between its reduced and the oxidized states as the peak potential difference is very close to 59 mv. The CV study for the PF as given in Figure 4 indicates a quasi-reversible behavior between its reduced and the oxidized states.

(1) $$\begin{array}{lll} {\text{C}}_{6}{\text{H}}_{12}{\text{O}}_{6}+\;6{\text{H}}_{2}\text{O}\to {\text{6CO}}_{2}+24{\text{H}}^{+}+24\text{e} & & {\text{E}}^{0}=0.014{\text{V}}_{\text{SHE}} \end{array}$$

(2) $$\begin{array}{lll} 6{\text{O}}_{2}+24{\text{H}}^{+}+24\text{e}\to {\text{12H}}_{2}\text{O} & & {\text{E}}^{0}=1.23{\text{V}}_{\text{SHE}} \end{array}$$

In the microbial fuel cell, glucose oxidation is the main source of energy generation. The individual potential of both the anode and the cathode depends on the redox potential of the reactions at the electrode. The oxidation half reaction of glucose, which is given in Equation 1, has a standard potential of 0.014 V, and theoretically, will determine the anode potential. However, it should be noted that the energy generated will not be available to extract directly at the electrode - since this conversion takes place in a microbe cell extraneous to the anode. On the other hand, the half reaction of the cathode, which is the reduction of molecular oxygen (Equation 2) will determine the potential of the cathode. The difference between the Equations 1 and 2 will give the fuel cell its theoretical maximum open circuit voltage of 1.216 V.

Under anaerobic conditions, *S. cerevisiae* undergoes fermentation where NADH oxidizes by alcohol dehydrogenase. However, some of the NADH will permeate outside the cell membrane. This will initiate a redox couple NADH / NAD^+^ on the periphery of the anode where an electron and a proton will be discharged (Equation 3) [7]. Due to the cellular membrane, the NADH transfer to the outside is significantly hindered. Therefore, the presence of a mediator attached to the membrane surface will greatly enhance the electron transfer to the electrode. The redox reaction that occurs within methylene blue is given in Equation 4.

(3) $$\begin{array}{ll} \text{NADH}\to {\text{NAD}}^{+}+{\text{H}}^{+}+{\text{e}}^{-} & {\text{E}}^{0}=0.01{\text{V}}_{\text{SHE}} \end{array}$$

(4) $$\begin{array}{ll} {\text{MB}}_{\text{(reduced)}}\to {\text{MB}}_{\text{(oxidized)}}+2{\text{e}}^{-}+{\text{H}}^{+} & {\text{E}}^{0}=-0{\text{.021V}}_{\text{SHE}} \end{array}$$

(5) $$\begin{array}{ll} {\text{PF}}_{\text{(oxidized)}}+{\text{e}}^{-}\to {\text{PF}}_{\text{(reduced)}} & {\text{E}}^{0}=0.361{\text{V}}_{\text{SHE}} \end{array}$$

The oxidation process of NADH is slow and inefficient [17]. In the case of yeast, NAD^+^/NADH redox couple would determine the anode potential, if no electron shuttling mediator was available. The use of MB as an intermediate molecule will be effective in this regard as it will get reduced relatively easily and will undergo a reversible reaction [18]. In the presence of a mediator molecule, the potential of the anode will be determined by the potential of the oxidation reaction given in Equation 4. In the six experiments conducted, the reactions at the anode and the cathode compartments are depicted in Table 2.

**Figure 5.**
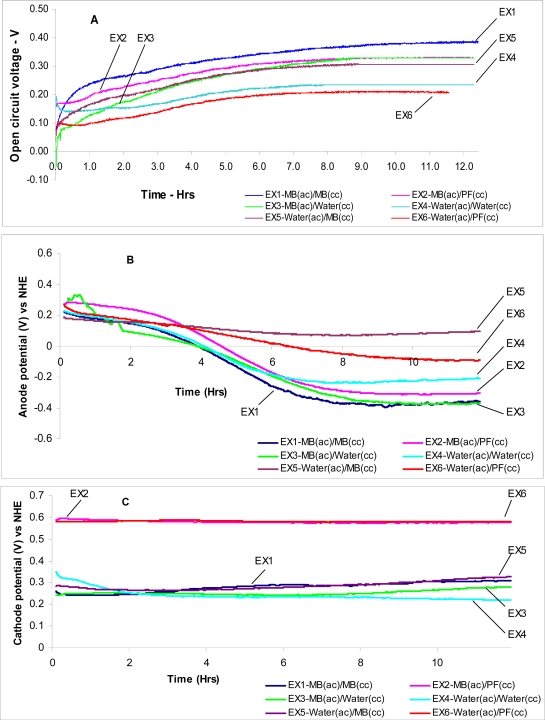
(A) Measured values of the cell open circuit voltage between anode and the cathode; (B) The potential variation in the anode; (C) The potential variation in the cathode. Cathode and anode potentials were measured against Ag/AgCl electrodes and subsequently converted to NHE by adding 0.197V; EX1 to EX6 are the samples prepared as given in Table (2); (ac- anode chamber, cc- cathode chamber); MB-methylene blue; PF - K_3_Fe(CN)6.

With respect to the reactions given in the Table 2, the theoretical open circuit potentials for each experiment are given in the Table 3. Figure 5 (A) shows the results that were obtained by direct measurement of the potential difference between the electrodes. It was evident that the direct measurement results were different from the values obtained by taking the difference of the individual anode and the cathode potentials. The possible explanation for this is the existence of a potential difference between the interfacing liquids in the cathode and anode compartments.

When there is no electron transfer molecule is present at the cathode, the reduction of oxygen takes place with a high overpotential. However, [Fe(CN)_6_^4–^/oxygen] coupled reaction and the (MB/oxygen) coupled reaction reduces the overpotential and makes it easier for molecular oxygen to get reduced. At the cathode, when either MB or Fe(CN)_6_^4–^ is present, the potential is determined by the redox reaction depicted in Equations 4 and 5. Figure 5 (B) represents the anode potential converted to the normal hydrogen electrode (NHE) potential values. The potential changed from positive to more negative with time, indicating that the anode oxidation reaction dominated. According to Figure 5(B), out of all experimental units, EX1 [MB(ac)/MB(cc)] exhibited the highest anode potential of 0.394±0.017 V. All the samples with MB in the anode compartment exhibited higher potentials, indicating that MB functioned as a superior mediator. When the anode compartment had no mediators the EX5 [water(ac)/MB(cc)] gave the lowest potential of 0.096±0.007 V.

On the cathode compartment, the potential values remained positive. It was observed that with time, the positive values increased implying that the redox reaction became dominant. Of all the experimental units, EX2 [MB(ac)/PF(cc)] and EX6 [water(ac)/PF(cc)] gave the highest cathode potentials with 0.578±0.002 V and 0.5810±001 V, respectively [Figure 5 (C)]. In both these cases, the cathode solution was K_3_Fe(CN)_6_. However, a relatively higher potential was recorded for EX6 [water(ac)/PF(cc)]. This indicated that the electrolyte of the opposite compartment had a significant effect on the potential of a particular electrode. For example, the anode potential of EX1 [MB(ac)/MB(cc)] and EX2 [MB(ac)/PF(cc)] were not the same even though the EX1 and EX2 anode solutions were similar. This is because the cathode solutions of the two experimental units were different. Similarly, the cathode potential of EX2 [MB(ac)/PF(cc)] and EX6 [water(ac)/PF(cc)] were not the same even though the cathode solutions of EX2 [MB(ac)/PF(cc)] and EX6 [water(ac)/PF(cc)] were similar. This behavior could be attributed to the differences of the anode solutions in the two experimental units. Another notable observation was migration of mediators across compartments - though at a relatively slower rate (as indicated by slow coloration of the opposite compartment when methyl blue was used in one compartment). This purports that even though the cell is compartmentalized, the membrane separating the chambers were not totally impermeable for the mediator molecules. The implication of this cross over is reduction of possible maximum voltage differences which in turn reduce the power capacity of the fuel cell.

### 3.2. Fuel Cell Behavior under Load

**Figure 6.**
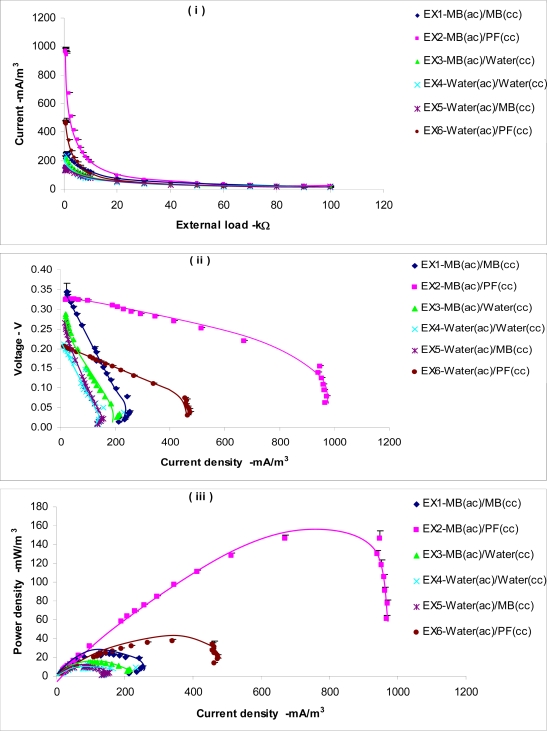
Variation of fuel cell parameters under different load conditions (i.) Power Vs Load, (ii) Load current Vs Load and (iii) Load voltage Vs Load.

The vitreous carbon electrode of 45.PPI imparts a large surface area exposed to the solution. Therefore, parameters like current and power was presented with respect to the electrode volume rather than the electrode surface area in the cell performance analysis. For testing the fuel cell under different loads, the cell was connected to a decade resistor box and the potential difference across the load was recorded. The cell was loaded under three load ranges, A, B and C as given in Table 4, where in each instance, the cell was loaded with an external resistor for 10 minutes. When loading the cell in the 100,000 – 10,000 Ω range, the potential drop was almost negligible. However, it was observed that as the load decreased, the current increased resulting in more losses. The effect of mediators used in the cathode is elucidated in Figure 6. The experimental pairs EX2 [MB(ac)/PF(cc)] and EX6 [water(ac)/PF(cc)], EX1 [MB(ac)/MB(cc)] and EX5 [water(ac)/MB(cc)], EX3 [MB(ac)/water(cc)] and EX4 [water(ac)/water(cc)] had identical cathode solutions. The behavior of voltage and current of each pair shows a significant similarity [see Figures 6 (ii)–6 (iii)] and it bears the proof that the cathode solution has a significant effect on the fuel cell parameters. In Figure 6(iii), the EX2 [MB(ac)/PF(cc)] showed the best performance in terms of power generation where 146.71±7.7 mW/m^3^ was measured at the load of 1000 Ω.

**Figure 7.**
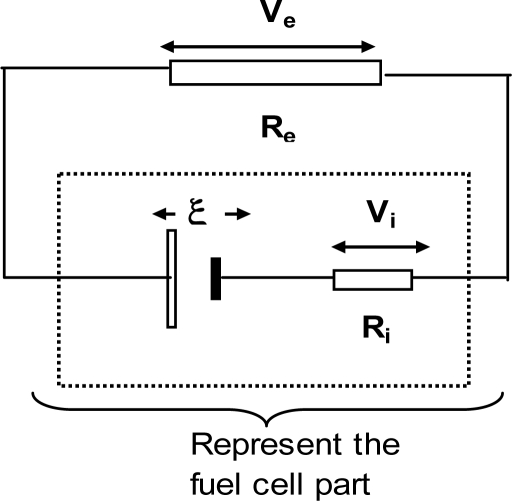
A model diagram for a fuel cell with R_i_ the internal impedance, ξ the OCV of the cell, and R_e_ the external load (V_e_ and V_i_ are the voltage drops across R_e_ and R_i_).

**Figure 8.**
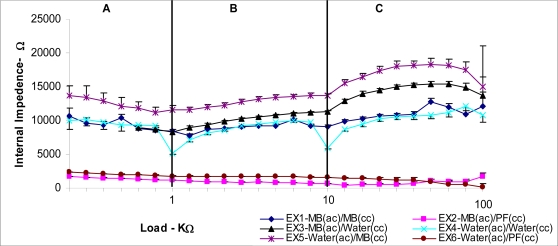
The fuel cell internal impedance variation under different loads

The internal impedance of the fuel cell has a significant effect in determining the voltage output. Impedance rather than the resistance is quite appropriate for a BFC as the cell has a capacitive component between the two electrodes [19]. According to the polarization graphs in Figure 6 (ii) the EX2 and EX6 both shows closer behavior to the ideal curve in the ohmic region. This explains the use of PF in the cathode as a mediator significantly reduces the ohmic loss compared to other mediator combinations. The fuel cell schematically can be represented with the internal impedance component Ri, as shown in Figure 7. The impedance values for EX2 [MB(ac)/PF(cc)] and EX6 [water(ac)/PF(cc)] were the lowest and were fairly stable over a wider range of loads as shown in Figure 8. All the other experimental units showed relatively higher impedance values varying significantly with the cell loading range. Since the electrodes used in the experiment were fairly large, it could be anticipated that the capacitive component is significantly large [19].

It was observed that the internal impedance was substantially influenced by the cathode solution. Presence of K_3_Fe(CN)_6_ in the cathode significantly reduced the internal impedance. This may be attributed to the presence and increased mobility of ions in the solution. On the other hand, experimental units containing MB in the catholite caused higher resistance values explaining further that cathode solutions should be ionic in order to lower the internal impedance. The lowest impedance recorded was approximately 454±26.8 Ωas per Figure 8 and it was obtained when the cell was loaded with 20 kΩin EX2 [MB(ac)/PF(cc)]. A current of 16.09±8 μA was recorded when the cell was operating under lowest impedance. It should be noted that in addition to the solution properties, the membrane and gap between the electrodes also made significant contributions to the internal impedance.

### 3.3. Fuel cell efficiency

The chemical efficiency of the fuel cell over the load range tested is dependant on the type of reaction the fuel (glucose) undergoes. Thermochemically, efficiency of glucose metabolism could be calculated as follows:

(6) Efficiency = ΔG/ΔH

where, ΔG_f_ - Gibbs free energy, ΔH – Enthalpy of formation of glucose.

For glucose, ΔG_f_ = –910KJ/mol, ΔH_f_ = –1268KJ/mol and according to Equation 6, the efficiency of energy conversion is 0.718. Accordingly, from its formation enthalpy, 71.8% of energy is available for use. The operational efficiency of a fuel cell is an important parameter. The Energy Efficiency-μ of the fuel cell is given by the Equation 7. where V is the voltage across the load ,ΔH is the calorific value of Glucose, n - is the number of electron moles transferred across the load and F- is the Faraday constant [20].

(7) $$\mu =\frac{V}{\Delta H/n*F}$$

**Figure 9.**
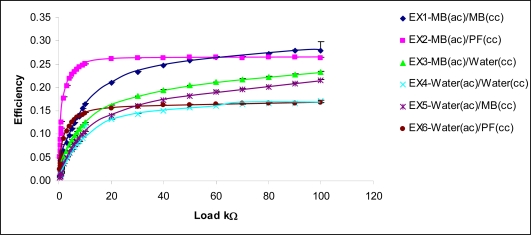
The efficiency variation of the fuel cell under varying external loads.

The efficiencies calculated for the six experiments are depicted in Figure 9. For higher load conditions or when the load currents were low, the cell showed higher efficiencies. At the best operating point where the maximum power was recorded (1000 Ω load), the efficiency for EX2 [MB(ac)/PF(cc)] was 12.6±0.2 %. The highest efficiency obtained from the fuel cell was 28±1.8 % for the EX1 at 100 KΩ. However, for EX2 [MB(ac)/PF(cc)], the efficiency value remained at 26.4% for a wide range of external loads. This fairly constant efficiency displayed by EX2 [MB(ac)/PF(cc)] could be attributed to the stable electron transfer between the mediator molecules and the electrode. However, when the current demand increases, with the decrease in external load, the electron generation and transfer at the anode is inefficient which results in a reduction of the load voltage. Consequently, the efficiency too will drop. In addition to the internal resistance, there is a capacitive effect building up near the electrodes which creates an additional potential drop between the fuel cell electrodes. This effect, known as double layer capacitance, was out of the scope of this experiment.

## 4. Concluding Remarks

*Saccharomyces cerevisiae,* an eukaryotic cell, performed favorably as the bio-catalyst in a glucose powered microbial fuel cell. The presence of methylene blue and the ferricyanide electronophores significantly enhanced the fuel cell performance. The cathode solution had a great influence on the potential difference across the cell terminals. The yeast microbial fuel cell generated a maximum power of 146.71±7.7 mW/m^3^ with a maximum open circuit voltage of 383.6±1.5 mV. The maximum operational efficiency of the fuel cell was 28±1.8 % which was obtained when methylene blue was the mediator in both anode and cathode compartments. MFCs tested with prokaryotic species such as *E-coli* with neutral red as the mediator has shown OCV of 850 mV and closed circuit potential of 620 mV for 120 Ω[21]. The relatively low current generation under different loads in the yeast fuel cell can be attributed to the slow metabolic rate of yeast compared to *E-coli.* Use of microorganisms that self-mediate electrons has produced outstanding results. With a microbial consortia consisting of *Alcaligenes faecali*, *Enterococcus Gallinarum, Pseudomonas aeruginosa* and other *Pseudomonas sp.* it has been able to obtain an electron transfer efficiency of 80% [22]. As the voltage efficiency for the yeast fuel cell was around 28%, it’s clear that the electron transfer efficiencies were comparatively low. Overall, the low performance of the fuel cell could be attributed to high over potential for reduction of O_2_ at the reticulated vitreous carbon cathode, and electron transfer inefficiencies between the mediator molecules and the microorganism’s cell wall. When fermentation takes place in the presence of an electrode not all the electrons are available in the electron donor for harvesting. Percentage wise, less than 60% would be recovered [12]. For the yeast fuel cell to be a viable power source, there are many challenges to overcome. Among them fully understanding the electron transfer kinetics with yeast metabolism and kinetics of the mediators are significant. Graphite electrode modified with neutral red has shown increased performance compared to a bare electrode [23]. Hence, the use of modified electrodes with a mediator (MB) in the yeast fuel cell would be an important aspect to consider. Numerous techniques exist for immobilization of organic molecules in order to functionalize an electrode [24, 25]. MB immobilized on a cellulose surface functionalized with TiO_2_/Ti_3_PO_4_ can be incorporated on to a carbon electrode. Studies have shown that Ti_3_PO_4_ enhanced electrochemical properties between the mediator and the electrode [25]. Future studies should target evaluating such techniques for performance enhancement of MB mediated yeast fuel cells.

## Figures and Tables

**Table 1.** The experimental design of the experiment.

Exp. Number	Organism	Substrate	Mediator- (anode)	Catholyte
EX1	Yeast	Glucose	Methylene blue	Methylene blue
EX2	Yeast	Glucose	Methylene blue	K_3_Fe(CN)_6_
EX3	Yeast	Glucose	Methylene blue	Water
EX4	Yeast	Glucose	Water	Water
EX5	Yeast	Glucose	Water	Methylene blue
EX6	Yeast	Glucose	Water	K_3_Fe(CN)_6_

**Table 2.** Fuel cell reactions for different experiments: (a) the reactions in the anode compartment and (b) the reactions in the cathode compartment.

(a)	
Experiment	Reactions in the anode compartment
EX1	MB_(oxidized)_+NADH_(yeast)_ → NAD^+^_(yeast)_ + MB_(reduced)_ MB_(reduced)_ → MB_(oxidized)_ + 2e^–^ + H^+^
EX2	MB_(oxidized)_+NADH_(yeast)_ → NAD^+^_(yeast)_ + MB_(reduced)_ MB_(reduced)_ → MB_(oxidized)_ + 2e^–^ + H^+^
EX3	MB_(oxidized)_+NADH_(yeast)_ → NAD^+^_(yeast)_ + MB_(reduced)_ MB_(reduced)_ → MB_(oxidized)_ + 2e^–^+ H^+^
EX4	NADH_(yeast)_ → NAD^+^_(yeast)_ + H^+^ + e^–^
EX5	NADH_(yeast)_ → NAD^+^_(yeast)_ + H^+^ + e^–^
EX6	NADH_(yeast)_ → NAD^+^_(yeast)_ + H^+^ + e^–^

**Table:** 

(b)	
Experiment	Reactions in the cathode compartment
EX1	4MB_(oxidized)_ + 4H^+^ + 8e^–^ → 4MB_(reduced)_ 4MB_(reduced)_ + O_2_ → 2H_2_O + 4MB_(oxidized)_
EX2	4PF_(oxidized)_ + 4e^–^ → 4PF_(reduced)_ 4PF_(reduced)_ + O_2_ + 4H^+^ → 2H_2_O + 4PF_(oxidized)_
EX3	4H^+^ + O_2_ + 4e^–^ → 2H_2_O
EX4	4H^+^ + O_2_ + 4e^–^ → 2H_2_O
EX5	4MB_(oxidized)_+4H^+^ + 8e^–^ → 4MB_(reduced)_ 4MB_(reduced)_ + O_2_ → 2H_2_O + 4MB_(oxidized)_
EX6	4PF_(oxidized)_ + 4e^–^ → 4PF_(reduced)_ 4PF_(reduced)_ + O_2_ + 4H^+^ →2H_2_O + 4PF_(oxidized)_

**Table 3.** The theoretical and practical open circuit voltage for each mediator combination.

Experiment No.	Open circuit Voltage-V (Theoretical)	Open circuit Voltage-V (Practical)
EX1	1.198	0.386
EX2	0.858	0.332
EX3	1.219	0.327
EX4	1.209	0.234
EX5	1.188	0.305
EX6	0.848	0.209

**Table 4.** The loading patterns for the cell.

Loading pattern	Range	Increment
A	400Ω–1000Ω	100Ω
B	1kΩ–10kΩ	1kΩ
C	10kΩ–100kΩ	10kΩ
